# 3D rainbow phononic crystals for extended vibration attenuation bands

**DOI:** 10.1038/s41598-020-75977-8

**Published:** 2020-11-04

**Authors:** H. Meng, N. Bailey, Y. Chen, L. Wang, F. Ciampa, A. Fabro, D. Chronopoulos, W. Elmadih

**Affiliations:** 1 Institute for Aerospace Technology & The Composites Group, The University of Nottingham, Nottingham, NG7 2RD UK; 2 Department of Mechanical Engineering, University of Louisville, Louisville, KY 40208 USA; 3 School of Mechanical Engineering, Xi’an Jiaotong University, Xi’an, 710049 China; 4 Department of Mechanical Engineering Sciences, University of Surrey, Guildford, GU2 7XH UK; 5 Department of Mechanical Engineering, University of Brasilia, Brasilia-DF, 70910-900 Brazil

**Keywords:** Mechanical engineering, Mechanical properties, Applied physics

## Abstract

We hereby report for the first time on the design, manufacturing and testing of a three-dimensional (3D) nearly-periodic, locally resonant phononic crystal (PnC). Most of the research effort on PnCs and metamaterials has been focused on the enhanced dynamic properties arising from their periodic design. Lately, additive manufacturing techniques have made a number of designs with intrinsically complex geometries feasible to produce. These recent developments have led to innovative solutions for broadband vibration attenuation, with a multitude of potential engineering applications. The recently introduced concept of rainbow metamaterials and PnCs has shown a significant potential for further expanding the spectrum of vibration attenuation in such structures by introducing a gradient profile for the considered unit cells. Given the above, it is expected that designing non-periodic PnCs will attract significant attention from scientists and engineers in the years to come. The proposed nearly-periodic design is based on cuboid blocks connected by curved beams, with internal voids in the blocks being implemented to adjust the local masses and generate a 3D rainbow PnC. Results show that the proposed approach can produce lightweight PnCs of a simple, manufacturable design exhibiting attenuation bandwidths more than two times larger than the equivalent periodic designs of equal mass.

## Introduction

Phononic crystals (PnCs) and metamaterials can be considered as engineered materials with designed macroscopic periodic or non-periodic structures that exhibit acousto/elastic responses not achievable in natural materials^1^. A universally accepted distinction between PnCs and metamaterials does not yet exist. Some researchers define metamaterials as artificially structured media showing novel acousto/elastic effects for wavelengths much greater than their periodicities. Others extend the concept of metamaterials to include PnCs.

PnCs with periodic structures are the mechanical analog of well-known photonic crystals in optics and have been studied since the pioneering work of Lord Rayleigh in 1887^2^. However, only recently, PnCs have received widespread interest because their design can be supported by modern optimizers with high computing performance and innovative additive manufacturing (3D-printing) processes, which provide unprecedented manufacturing freedom for the fabrication of intricate parts and geometries.

From a structural viewpoint, PnCs are an arrangement of many elementary cells, each containing an inclusion or a cavity inducing non-conventional dispersion properties^3^. Depending upon their geometry and constituents, PnCs include: (a) structures made of two or more materials with different mechanical properties in a periodic repetition, (b) structures with enclosed periodic units such as holes containing fluids and (c) waveguide structures characterised by periodic surface profiles^4–7^. A distinguishing peculiarity of PnCs is that they can be designed and manufactured to inhibit the propagation of acoustic/elastic waves in specific frequency ranges known as phononic band gaps or stop bands^8^. Outside these band gaps, waves can propagate in any direction. So, PnCs can be considered as material-enabled signal filters. This property allows PnCs to be used for frequency-dependent control of the trajectory and dissipation characteristics of acoustic and stress waves^9^. As a result, PnCs have found natural applications in research areas such as acoustic lenses^10^, wave filters^11^, frequency modulators^5^, acoustic cloaks^12^, and thermal insulators^4^.

The two broadly accepted physical mechanisms at the origin of phononic band gaps are the Bragg-scattering and local resonance effects^3,13^. In the former mechanism, stop bands are generated by the destructive interference of both incident and reflected waves in specific frequency bands, which are excited resonantly when the wave-vector is equal to 2 $\pi$ /$\Lambda$, where $\Lambda$ is the periodicity of the PnC structures. The Bragg-scattering effect enables phononic band gaps with broader frequency ranges. However, Bragg-scattering stop bands strongly depend on the geometry of the lattice and the shape of scattering. In the local resonance effect, instead, stop bands are induced by local resonances of the internal oscillating inclusions of the PnC^3^. However, the width of a local resonant band gap is typically narrow^14,15^.

A theoretical and experimental study of longitudinal wave propagation in a rod structure including periodic PnC local resonators was presented by Wang et al.^16^. The authors showed that the band gap attenuation was influenced by the stiffness and mass ratios of the local resonator. However, their work did not fully explore the band gap formation mechanism. A more systematic study was provided by Xiao et al.^17^, who investigated stop bands of metamaterial rods combined with periodically attached local resonators through a Finite Element (FE) approach, their work showed that the wave attenuation is efficient only at the resonator eigen frequencies and dramatically decreases away from them^18^.

That is why regardless of numerous PnCs proposed in recent decades, few have been shown to possess simultaneously wide and robust stop bands^19^. Hence, a broadband control of low-frequency waves using lightweight structures remains a challenge. This consideration led to the new three-dimensional (3D) rainbow-trapping design. Originally proposed in the context of optical waves^20^, this concept has been further developed for acoustic and elastic waves. Zhu et al.^21^ have proposed the first experimental demonstration of acoustic rainbow trapping, i.e. a metamaterial containing non-periodic grooves that trap broadband acoustic waves. Chen et al.^22^ have applied this concept to a gradient metamaterial beam to observe the enhancement of flexural waves in beams. Also, Beli et al.^23^ have experimentally shown that the wave trapping effect can also arise from manufacturing variability in printed metamaterials and that the mistuning of the resonators can also prevent the band formation, thus requiring careful design of the spatial profile of the rainbow metamaterial. This effect is shown to be more relevant in Bragg-scattering of resonators^24^. Moreover, it has been shown that both spatially graded and random arrangements result in bandgap widening in locally resonant metamaterials^25^. Recently, Meng et al.^26^ have proposed a multi-frequency broadband rainbow metamaterial with one-dimensional bandgaps. Meng et al.^27^ have also shown that optimal spatial distribution of the tuning frequency of each resonator can be further explored for different performance criteria in multi-frequency metamaterials, thus opening new and innovative possibilities for metamaterial design.

Motivated by these results, this report investigates the attenuation performance of a novel 3D rainbow PnC produced using addtive manufacturing (AM) processes. The proposed design is based on spatially distributed cubic blocks connected by two curved beams. Internal holes in the cubic block tessellated in the structural design are used to adjust the local masses and create a spatial gradient profile. Consequently, broadband attenuation can be achieved for *x*, *y* and *z* directions in the structure, i.e. a 3D attenuation band, as a result of the rainbow PnC design. It is later shown, by experimental and numerical results, that the proposed approach can produce PCs with attenuation bandwidths more than two times larger than the periodic design with the same mass. Practical aspects of the employed AM technology are also discussed.

## Results and discussion

**Figure 1:**
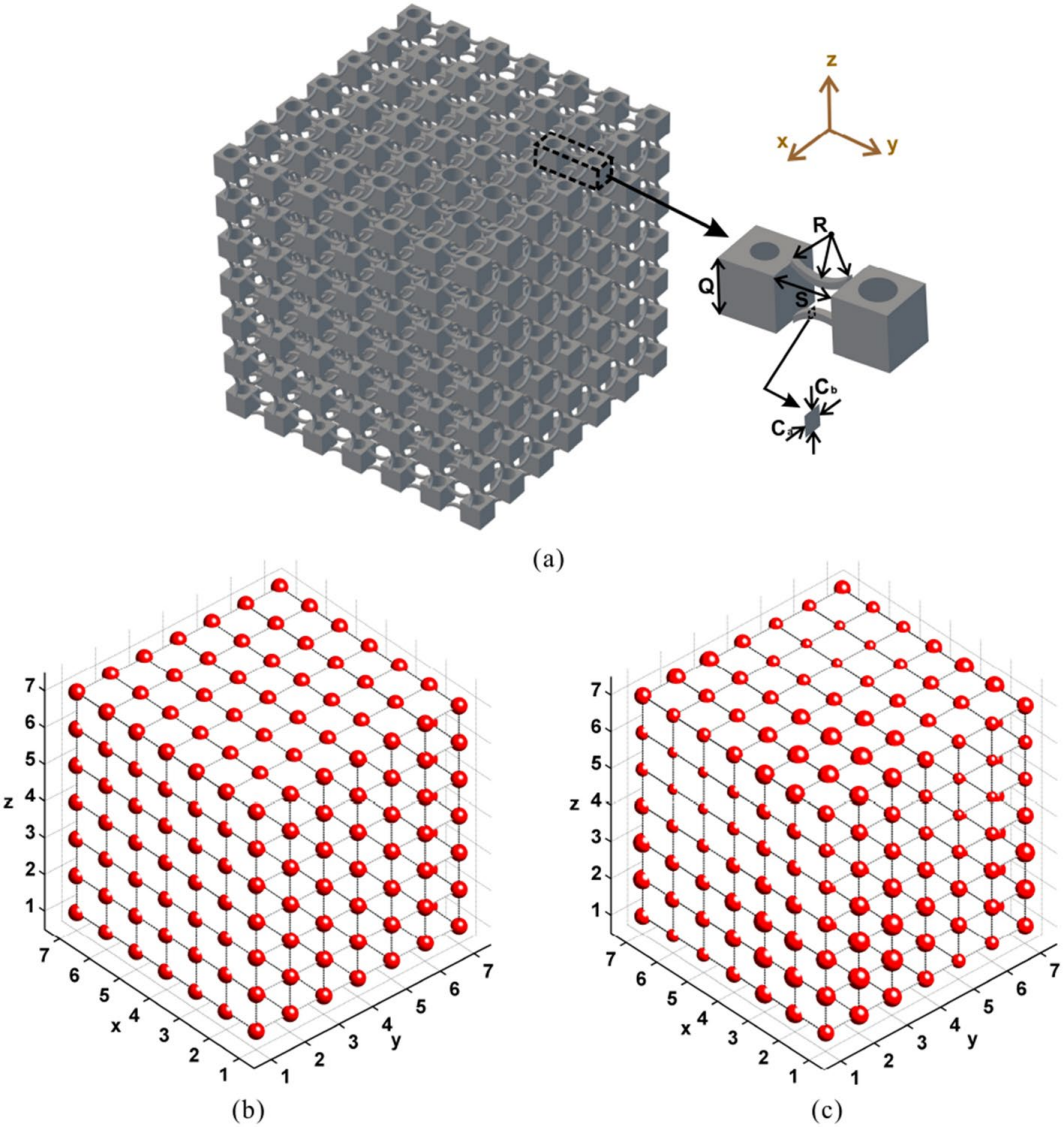
Design of the investigated 3D PnC: (**a**) schema of the 3D rainbow PnC, the labels show the geometric parameters block side length (*Q*), blocks distance (*S*), radius of the curved beam (*R*), cross-section dimensions of the curved beams ($C_{a}$, $C_{b}$). Block mass distributions ($m_{ijk}$) of (**b**) periodic and (**c**) rainbow PnCs by bubbles of different dimensions.

To explore the effect of non-periodicity on the frequency response functions (FRFs), 3D PnCs structures consisting of spatially distributed blocks connected by two curved struts as shown in Fig.1a are investigated. To deliver the rainbow structural design, the mass of each block is changed by opening a cylindrical hole in the middle of it which would allow for a mass reduction between 0% and 70%. This upper limit is set in order to preserve the structural integrity of the blocks and to maintain the assumption that blocks act as rigid bodies. The masses of blocks are set as sinusoidally varying over the *x*, *y* and *z* periodicity directions of the rainbow structure with the aim of inducing a smooth near-periodicity and avoiding rapid mechanical impedance changes for consecutive masses. The two curved struts between blocks are inverted towards each other. Curvature can both enhance stability and reduce transmission rigidity compared to straight beams. The cross-section of the connecting beams and consequently connecting stiffness is fixed. The equivalent periodic structure is straightforward to derive by calculating the diameter of a periodic set of holes in the vibrating masses that would induce a structure of equal mass to the rainbow one.

The dynamic properties of the rainbow PnCs were investigated both numerically and experimentally. The material has a density of 1000 kg/m^3^, Young’s modulus of 1.75 GPa, and Poisson’s ratio of 0.35. The proposed rainbow PnC structure has a periodicity of seven in 3D (i.e. forming a 7 × 7 × 7 rainbow PnC). The side length of cubic blocks is *Q* = 20 mm, the distance between blocks is *S*=20mm, the radius of the curved beam is *R* = 20 mm and the cross-section dimensions of the curved beams are $C_a$=$C_b$= 2.5 mm. The mass *m* of the rainbow PnC can be calculated using the following sinusoidal distribution in 3D: $m_{ijk}=(0.675+0.115\sin ((i-1)\pi /3)+0.115\sin ((j-1)\pi /3)+0.115\sin ((k-1)3\pi /3))M$, where *M* is the mass of original non-opening blocks and *i*, *j*, *k* = 1,2,…,7 represent the number of cuboid blocks in *x*, *y* and *z* directions. The block mass of corresponding periodic PnC is equal to the average block mass of the rainbow PnC and is given by: $m_{ijk}=0.675M$. The block mass distributions of periodic and rainbow PnCs are depicted in Fig. 1b and c.

A FE approach was employed to compute the FRFs of the 3D nearly-periodic PnCs. Elastic PnCs and metamaterials can be modelled by analytical and numerical methods, such as plane-wave expansion methods^28–31^, finite-difference time-domain^32–35^, layer-multiple scattering method^36–38^ and FE approaches^39–42^. Compared to other modelling schemes with a range of limitations, the FE method is the most pertinent one for non-periodic structures and able to elaborate accurately on their internal dynamics and interactions. In the FE modelling, forces in *x*, *y* and *z* directions are exerted on one vertex of the PnCs and displacements of the diagonal vertex are then computed, with which the receptances of the structures in all directions can be obtained by $R_x=20\log 10|D_x/F_x|$, $R_y=20\log 10|D_y/F_y|$, $R_z=20\log 10|D_z/F_z|$, where $F_x$, $F_y$, and $F_z$ are the excitation forces, and $D_x$ , $D_y$, and $D_z$ are the acquired displacements in the three directions. More details of the FE modelling scheme are given in the “Methods” section.

**Figure 2:**
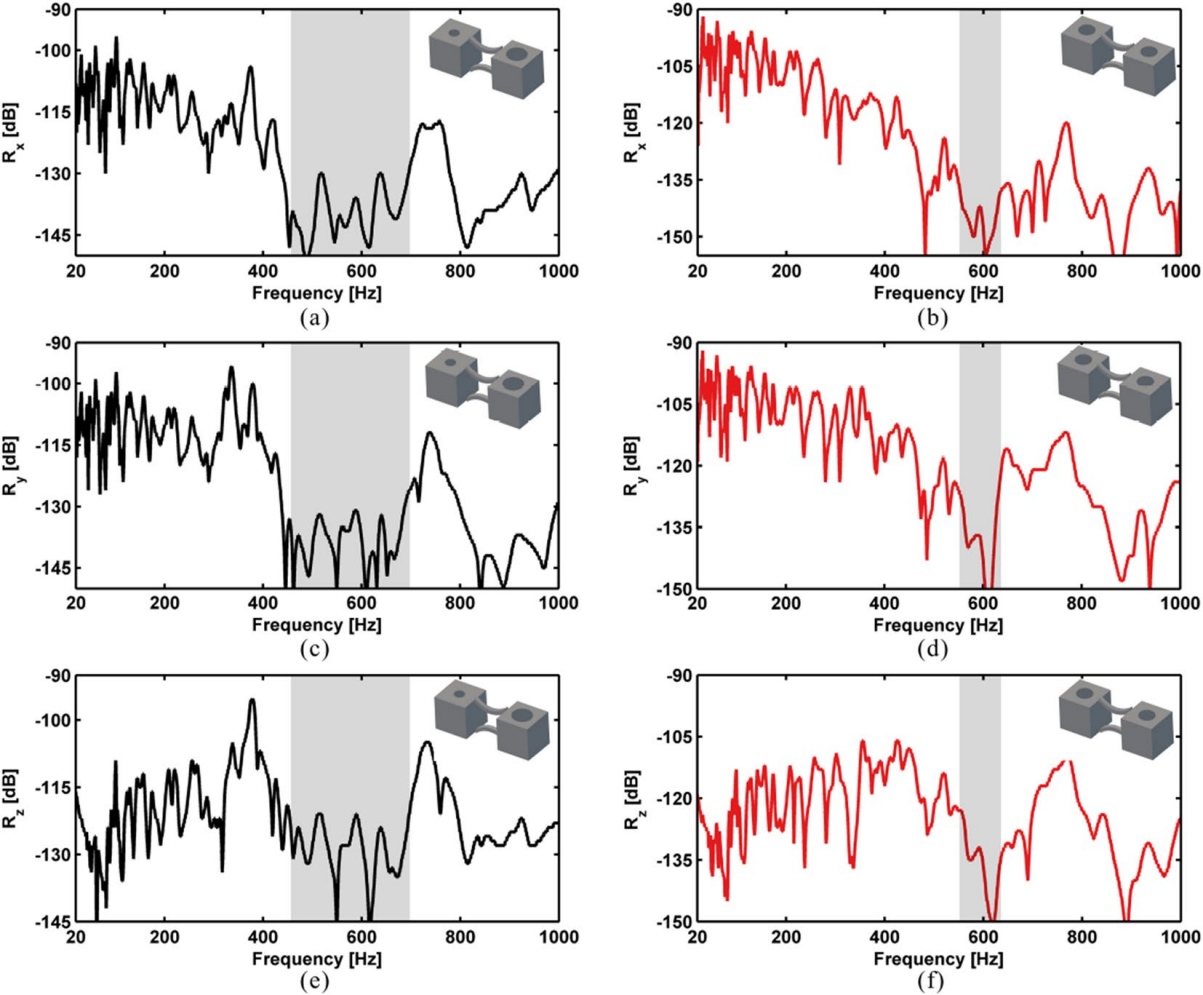
FE simulated displacement FRFs for the rainbow PnC in (**a**) *x*, (**c**) *y* and (**e**) *z* directions. Simulated response for the periodic PnC in (**b**) *x*, (**d**) *y* and (**f**) *z* directions. The stopbands are marked within grey areas.

**Figure 3:**
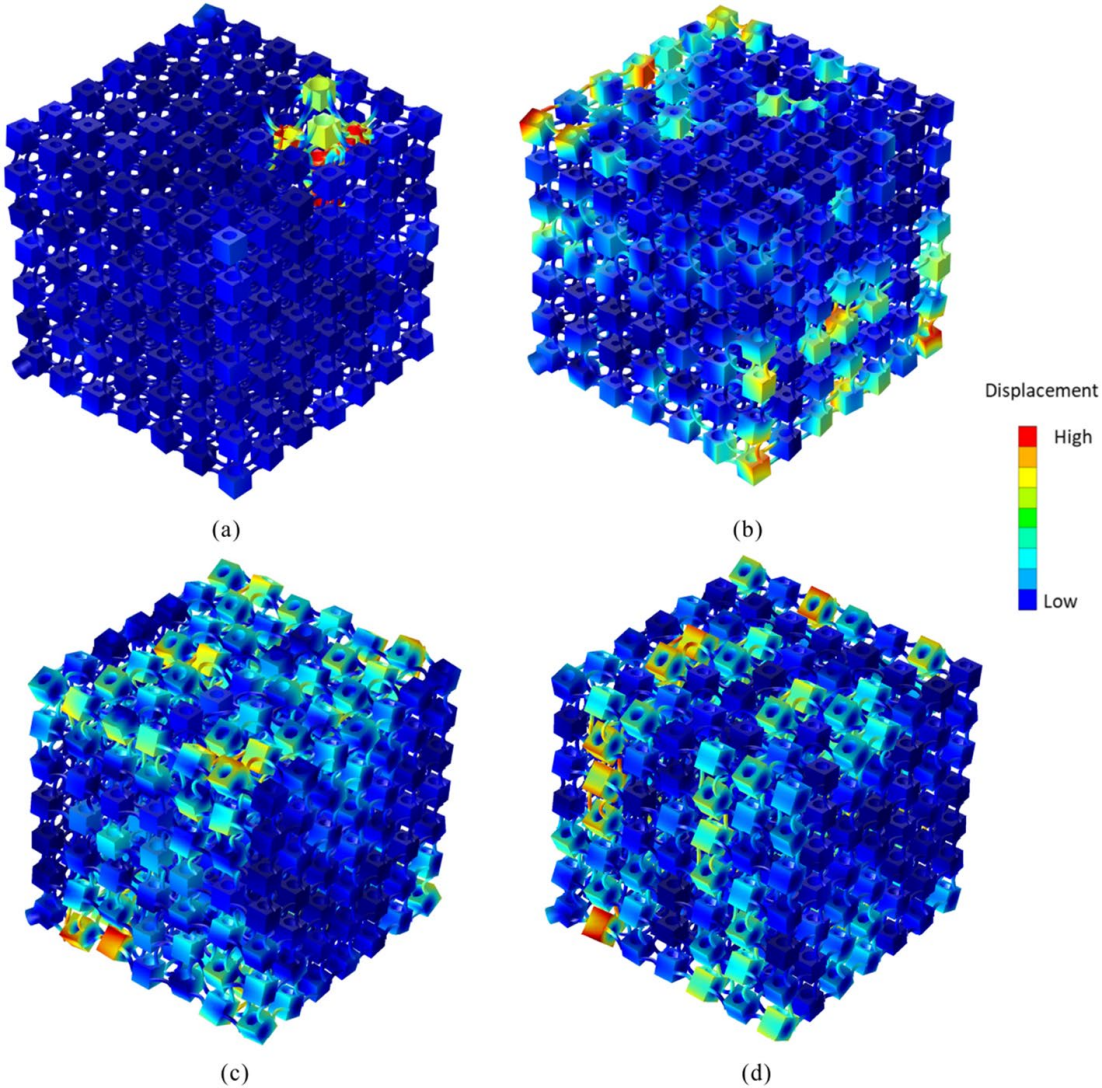
Mode shapes of PnCs at the starting and ending frequencies of the bandgaps: rainbow PnC at (**a**) 452 Hz and (**b**) 698 Hz, periodic PnC at (**c**) 550 Hz and (**d**) 639 Hz.

The calculated receptance values of the rainbow and periodic PnCs are shown in Fig.2a–f. It can be seen that the rainbow PnC has broader bandgaps than that of the periodic structure of the same mass . The bandgaps of the periodic PnC occur at the frequency range 550–638 Hz, within which the receptance values are significantly reduced. The mode shapes of the periodic PnC at the starting and ending frequencies of the bandgaps are shown in Fig. 3c and d. It can be seen that the bandgaps are formed by local resonance of the mass blocks. Wavelengths at bandgaps are also much larger than the lattice dimensions of the PnC. In contrast, the rainbow PnC has bandgaps within the frequencies 452–698 Hz. Mode shapes of the rainbow PnC at the starting and ending frequencies of the bandgap are shown in Fig. 3a and b. The rainbow PnC has enhanced bandgaps more than two times broader than that of periodic lattice. The extension of bandgaps should be attributed to the block mass variations in the rainbow PnCs, which will bring in local resonance at different frequencies. Meanwhile, it can also be observed that the reduction of receptance values within the bandgaps of the rainbow PnC is smaller than that of the periodic PnC. As discussed by Meng et al.^26^, the enlarged bandwidth could simultaneously induce smaller attenuation amplitude and thus smaller reduction of receptance values.

**Figure 4:**
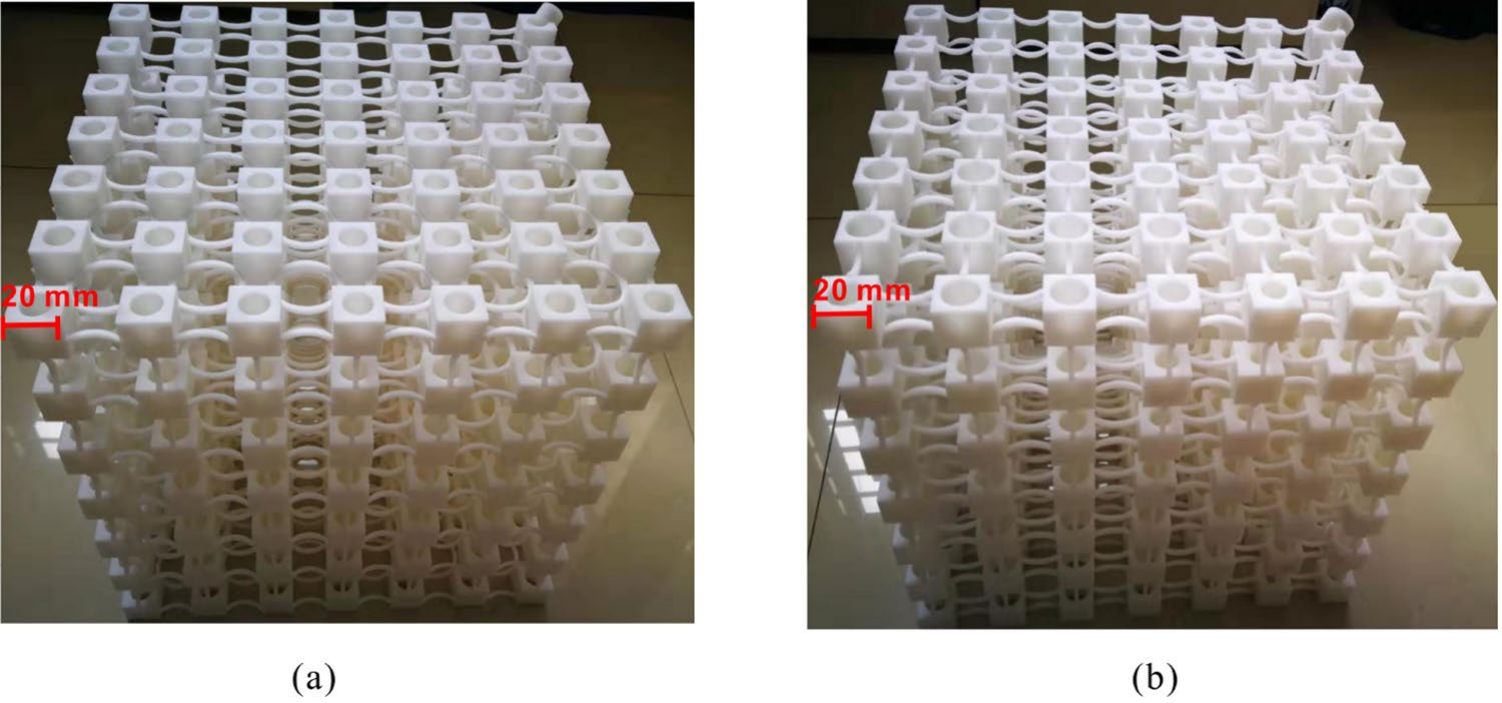
Photographs of the manufactured (**a**) periodic PnC and (**b**) rainbow PnC by laser sintering.

With the purpose of exploring further the effects of non-periodicity and verifying the above mentioned numerical results, experimental analysis was conducted on a rainbow and a periodic PnCs as shown in Fig. 4a and b. The two prototypes were realised through AM process. AM has become one of the most popular manufacturing methods for PnCs and metamaterials. It is more time-saving and less costly for the generation of objects with complex geometric shapes and enhanced precision than traditional subtractive manufacturing processes^26,27,43–48^. Numerous AM technologies have been employed for PnCs, including laser sintering, binder jetting and stereolithography^49,50^. Given the geometric dimensions, the structural physical parameter requirements, as well as the factor of cost, a Selective Laser Sintering (SLS) method was selected for the fabrication of the 3D PnCs. In-depth description of the fabrication process can be found in the “Methods” section.

The geometric parameters of the structures are identical to the ones employed in the numerical calculation. It should be stressed that 7 unit cells in each dimensions is considered as a low to moderate number for vibration stopband generation. Increasing the number of unit cells^51^ would imply deeper stopbands within the measured FRF structures. The choice of 7 unit cells resulted as a compromise between the maximum available dimensions for manufacturing the PnCs through AM (27 cm per direction). Besides, it is noted that an oblique cylinder was printed on the vertex of PnCs for the purpose of adhering the testing system which would exert the required excitation force on the PnCs. This oblique cylinder was also numerically modelled within the computations exhibited above.

**Figure 5:**
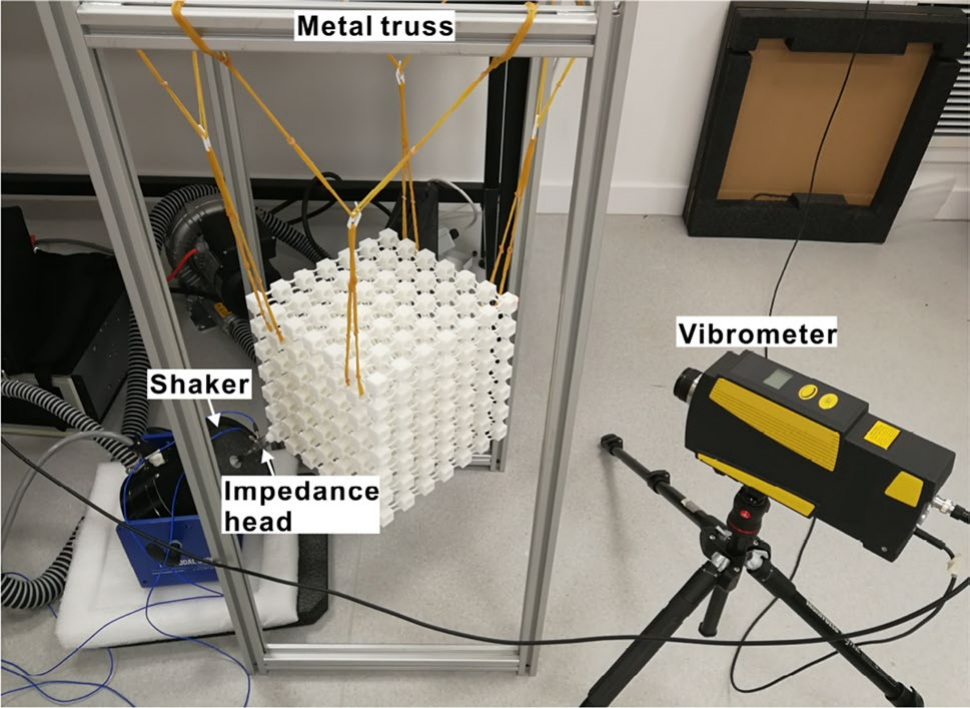
Experimental setup for dynamic testing of the 3D PnCs.

The samples were tested through the experimental setup presented in Fig. 5. A mechanical shaker was employed to excite the PnCs on the end face of the oblique cylinder with an input signal over the frequency range of 20–1000 Hz. The excitation forces on the PnCs were tested by an impedance head which was bolted to the mechanical shaker on the opposite end. Displacements in three directions at the diagonal corner of the excitation point were measured with the laser Doppler vibrometer. Due to the large dimensions and masses of the structures, the PnCs were suspended on a metallic truss using rubber bands and plastic hooks to simulate free boundary conditions. The approach of suspending the tested structures has been discussed and proved effective by Elmadih et al.^41,52–54^. More details of the experiment process are described in the “Methods” section.

**Figure 6:**
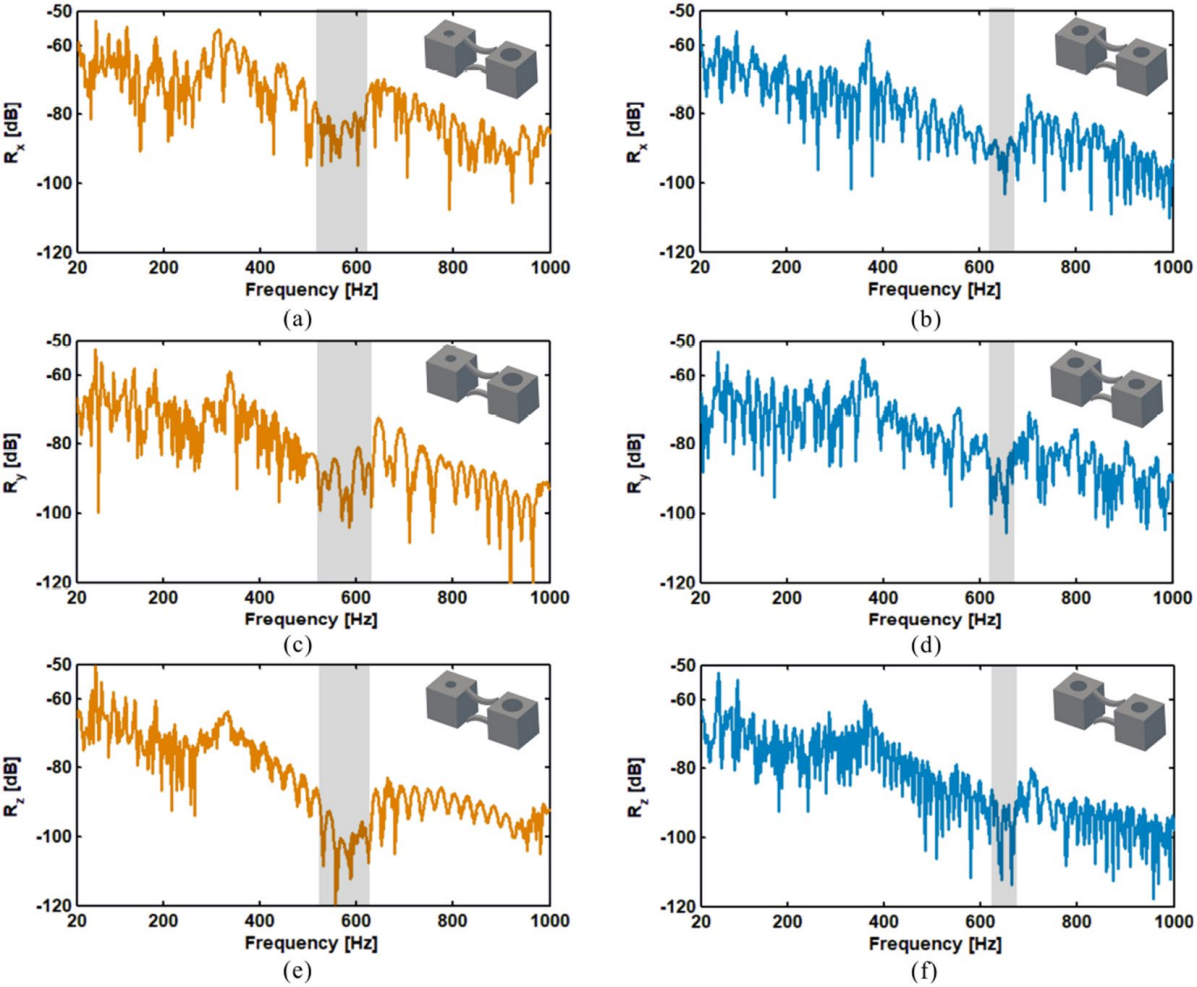
Experimental displacement FRFs of the rainbow PnC in *x* (**a**), *y* (**c**) and *z* (**e**) directions, and the periodic PnC in *x* (**b**), *y* (**d**) and *z* (**f**) directions.

The measured receptance values of the two samples are shown in Fig.6a–f. It can be seen that the experimental results display the same trends as the numerical ones. The rainbow and periodic PnCs have evident vibration attenuation in the frequency ranges of circa 515–620 Hz and 620–670 Hz respectively, as marked in grey areas in Fig.6a–f. The bandgaps of rainbow PnC are thus more than two times broader than those of the equivalent periodic PnC. It should be noted that the measured receptance values have reasonable differences in bandgap frequencies and bandwidths compared with the simulated results shown in Fig. 2a–f, which might be caused by the non-ideal experimental conditions and simulation models, especially the uncertainties introduced by the AM process. It has been found that the physical parameters (density, elastic modulus, etc.) in structures printed by AM technologies could have variabilities of up to more than 10%, which would change the bandgap frequencies and bandwidths of PnCs and metamaterials remarkably^23,46^. Despite the reasonable differences, both the experimental and numerical results proved the design method of enlarged bandgaps due to nonperiodic design, which could inspire future researchers to produce structures with enhanced vibration attenuation.

## Methods

### Finite element modelling

**Figure 7:**
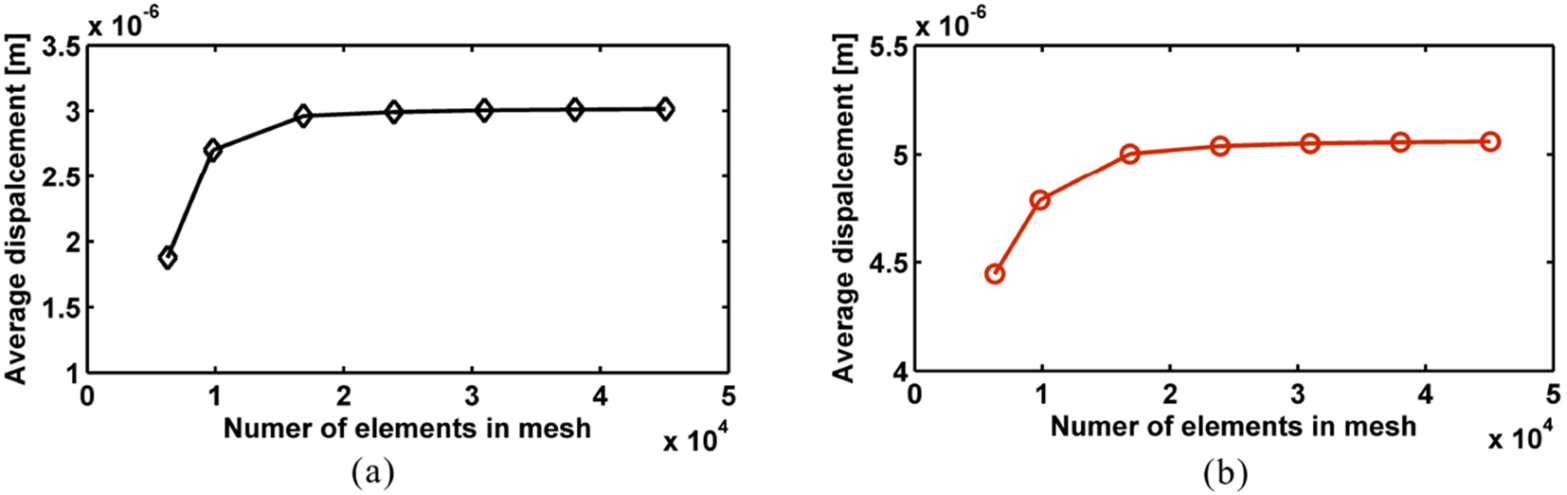
Mesh convergence study of finite element modelling with different element numbers with respect to the average displacement at the sampling point in the considered frequency range for (**a**) Rainbow PnC and (**b**) Periodic PnC.

A commercial FE tool was employed in order to numerically model the 7x7x7 structures. Due to non-periodicity, unit cell computational approaches were not applicable and the complete structure had to be modelled to simulate its broadband harmonic response. Linear hexahedra elements were employed for modelling the geometry of the structures. A mesh convergence study was performed in order to ensure accuracy and stability of the obtained results with a convergence criterion of 0.1% being employed for the maximum divergence between two mesh configurations as shown in Fig. 7a and b. A global damping lost factor of 0.03 was considered throughout the simulation process. The structural boundary conditions were assumed to be free-free with no structural displacement conditions imposed in order to emulate the physical experiment to a maximum degree. The input force was a 1 N force applied in a broadband spectrum between 0 and 1000 Hz. Each broadband frequency analysis consisted of 2000 steps in total with a total running time of 3830 s on a 2.2 GHz processor with 8 GB of RAM memory.

### Additive manufacturing technology employed

The structural samples were fabricated by a Laser Sintering 3D printer using PA12 material. The 3D printer employed a CO_2_ laser having a scan speed and hatch spacing of 0.35 m/s and 0.3 mm, respectively. The nominal spot size of the laser was 0.46 mm and the layer thickness 0.12 mm. A powder bed with dimensions of 375 mm × 375 mm × 430 mm was filled with PA12 powder at a temperature of 170. The accuracy of the printed geometries was related to the laser spot size, the layer thickness, the size of the powder and the laser scanning strategy etc. The minimum feature size by the 3D printer was 1 mm, below which considerable mechanical properties would be introduced owing to the existence of unsolidified powder^55^. The Young’s modulus and material density of the printed structures were measured on small fabricated samples based on the ASTM standard^56^ and found to be 1.75 GPa and 1000 kg/m^3^ respectively.

### Experimental process

**Figure 8:**
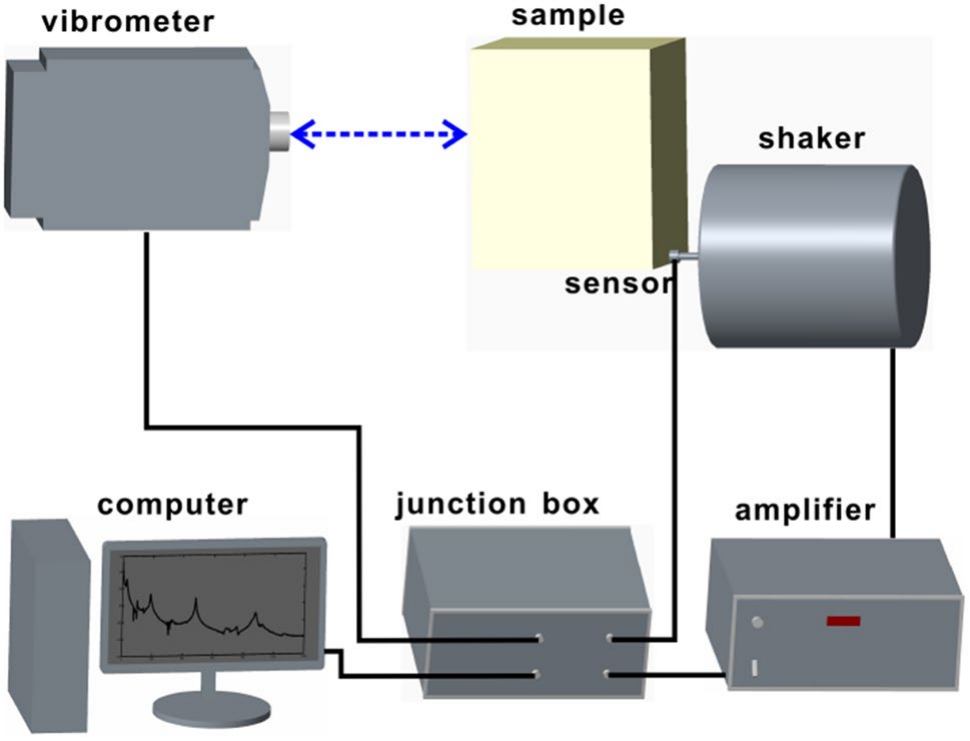
Caption of the experimental setup exhibiting connectivity between the signal generator, amplifier, structure under investigation, laser vibrometer and the signal acquisition unit.

During the experiment, a chirp wave with the frequency range of 20–1000 Hz was used as excitation signals. It was first generated by the computer and sent to a junction box (Polytec VIB-E-400), and afterwards amplified by a Modal Shop 2050E09 unit. The mechanical shaker (Modal Shop 2060E) excited the sample according to the received signals. An impedance head (PCB 288D01) mounted on the shaker was applied to test the force signals at the exciting point and a laser Doppler vibrometer (Polytec PSV-400) employed to measure displacement signals at sampling points. The collected displacements and forces were sent back to the junction box and calculated by the software to solve out the FRFs. All measurements were taken with a frequency resolution of 1.25 Hz and are complexly averaged over 100 spectral sweeps. The schematic illustration of the experiment setup is shown in Fig. 8.

## References

[1] Lu MH, Feng L, Chen YF (2009). Phononic crystals and acoustic metamaterials. Mater. Today.

[2] Rayleigh LXVII (1887). On the maintenance of vibrations by forces of double frequency, and on the propagation of waves through a medium endowed with a periodic structure. Lond. Edinb. Dublin Philos. Mag. J. Sci..

[3] Liu Z (2000). Locally resonant sonic materials. Science.

[4] Maldovan M (2013). Sound and heat revolutions in phononics. Nature.

[5] Cheng W, Wang J, Jonas U, Fytas G, Stefanou N (2006). Observation and tuning of hypersonic bandgaps in colloidal crystals. Nat. Mater..

[6] Poshakinskiy AV, Poddubny AN (2017). Phonoritonic crystals with a synthetic magnetic field for an acoustic diode. Phys. Rev. Lett..

[7] Ciampa F, Mankar A, Marini A (2017). Phononic crystal waveguide transducers for nonlinear elastic wave sensing. Sci. Rep..

[8] Liang B, Guo XS, Tu J, Zhang D, Cheng JC (2010). An acoustic rectifier. Nat. Mater..

[9] Yang S (2004). Focusing of sound in a 3D phononic crystal. Phys. Rev. Lett..

[10] Zhu J (2011). A holey-structured metamaterial for acoustic deep-subwavelength imaging. Nat. Phys..

[11] Elnady T (2009). Quenching of acoustic bandgaps by flow noise. Appl. Phys. Lett..

[12] Fleury R, Monticone F, Alù A (2015). Invisibility and cloaking: origins, present, and future perspectives. Phys. Rev. Appl..

[13] Sorokin VS (2016). Effects of corrugation shape on frequency band-gaps for longitudinal wave motion in a periodic elastic layer. J. Acoust. Soc. Am..

[14] Kuo NK, Piazza G (2011). Fractal phononic crystals in aluminum nitride: an approach to ultra high frequency bandgaps. Appl. Phys. Lett..

[15] Wang YF, Wang YS, Su XX (2011). Large bandgaps of two-dimensional phononic crystals with cross-like holes. J. Appl. Phys..

[16] Wang G, Wen X, Wen J, Liu Y (2006). Quasi-one-dimensional periodic structure with locally resonant band gap. J. Appl. Mech..

[17] Xiao Y, Wen J, Wen X (2012). Longitudinal wave band gaps in metamaterial-based elastic rods containing multi-degree-of-freedom resonators. N. J. Phys..

[18] Krushynska AO, Kouznetsova VG, Geers MG (2014). Towards optimal design of locally resonant acoustic metamaterials. J. Mech. Phys. Solids.

[19] Jia Z, Chen Y, Yang H, Wang L (2018). Designing phononic crystals with wide and robust band gaps. Phys. Rev. Appl..

[20] Tsakmakidis, K. L., Boardman, A. D. & Hess, O. ‘Trapped rainbow’ storage of light in metamaterials. *Nature* **450**, 397–401. 10.1038/nature06285 (2007). 10.1038/nature06285 18004380

[21] Zhu J (2013). Acoustic rainbow trapping. Sci. Rep..

[22] Chen YY, Zhu R, Barnhart MV, Huang GL (2016). Enhanced flexural wave sensing by adaptive gradient-index metamaterials. Sci. Rep..

[23] Beli D, Fabro AT, Ruzzene M, Arruda JRF (2019). Wave attenuation and trapping in 3d printed cantilever-in-mass metamaterials with spatially correlated variability. Sci. Rep..

[24] Achaoui Y, Laude V, Benchabane S, Khelif A (2013). Local resonances in phononic crystals and in random arrangements of pillars on a surface. J. Appl. Phys..

[25] Celli P, Yousefzadeh B, Daraio C, Gonella S (2019). Bandgap widening by disorder in rainbow metamaterials. Appl. Phys. Lett..

[26] Meng H, Chronopoulos D, Fabro A, Elmadih W, Maskery I (2020). Rainbow metamaterials for broadband multi-frequency vibration attenuation: Numerical analysis and experimental validation. J. Sound Vib..

[27] Meng H, Chronopoulos D, Fabro AT, Maskery I, Chen Y (2020). Optimal design of rainbow elastic metamaterials. Int. J. Mech. Sci..

[28] Qian D, Shi Z (2017). Using pwe/fe method to calculate the band structures of the semi-infinite beam-like pcs: Periodic in z-direction and finite in x-y plane. Phys. Lett. A.

[29] Hsu J-C, Wu T-T (2006). Efficient formulation for band-structure calculations of two-dimensional phononic-crystal plates. Phys. Rev. B.

[30] Wu F, Liu Z, Liu Y (2002). Acoustic band gaps created by rotating square rods in a two-dimensional lattice. Phys. Rev. E.

[31] Zhang X, Liu Z, Liu Y, Wu F (2003). Elastic wave band gaps for three-dimensional phononic crystals with two structural units. Phys. Lett. A.

[32] Sigalas M, Garcıa N (2000). Theoretical study of three dimensional elastic band gaps with the finite-difference time-domain method. J. Appl. Phys..

[33] Sigalas M, Garcıa N (2000). Importance of coupling between longitudinal and transverse components for the creation of acoustic band gaps: The aluminum in mercury case. Appl. Phys. Lett..

[34] Tanaka Y, Tomoyasu Y, Tamura S-I (2000). Band structure of acoustic waves in phononic lattices: Two-dimensional composites with large acoustic mismatch. Phys. Rev. B.

[35] Dawood A (2013). Finite difference time-domain modelling of metamaterials: Gpu implementation of cylindrical cloak. Adv. Electromagn..

[36] Liu Z, Chan C, Sheng P, Goertzen A, Page J (2000). Elastic wave scattering by periodic structures of spherical objects: Theory and experiment. Phys. Rev. B.

[37] Mei J, Liu Z, Shi J, Tian D (2003). Theory for elastic wave scattering by a two-dimensional periodical array of cylinders: An ideal approach for band-structure calculations. Phys. Rev. B.

[38] Psarobas I, Stefanou N, Modinos A (2000). Scattering of elastic waves by periodic arrays of spherical bodies. Phys. Rev. B.

[39] Wang Y-F, Wang Y-S (2013). Complete bandgap in three-dimensional holey phononic crystals with resonators. J. Vib. Acoust..

[40] D’Alessandro, L., Ardito, R., Braghin, F. & Corigliano, A. Low frequency 3d ultra-wide vibration attenuation via elastic metamaterial. *Sci. Rep.* **9**, 1–8 (2019). 10.1038/s41598-019-44507-6 PMC6541607 31142751

[41] Zhang H, Xiao Y, Wen J, Yu D, Wen X (2015). Flexural wave band gaps in metamaterial beams with membrane-type resonators: theory and experiment. J. Phys. D.

[42] Meng, H., Chronopoulos, D. & Fabro, A. T. Numerical simulation data for the dynamic properties of rainbow metamaterials. *Data Brief* **28**, (2020). 10.1016/j.dib.2019.104772 PMC6909204 31871966

[43] Li G-H, Wang Y-Z, Wang Y-S (2019). Active control on switchable waveguide of elastic wave metamaterials with the 3d printing technology. Sci. Rep..

[44] Lucklum F, Vellekoop MJ (2017). Design and fabrication challenges for millimeter-scale three-dimensional phononic crystals. Crystals.

[45] McGee O (2019). 3d printed architected hollow sphere foams with low-frequency phononic band gaps. Addit. Manuf..

[46] Fabro AT, Meng H, Chronopoulos D (2020). Uncertainties in the attenuation performance of a multi-frequency metastructure from additive manufacturing. Mech. Syst. Signal Process..

[47] Matlack KH, Bauhofer A, Krödel S, Palermo A, Daraio C (2016). Composite 3d-printed metastructures for low-frequency and broadband vibration absorption. Proc. Natl. Acad. Sci..

[48] Zheng X (2016). Multiscale metallic metamaterials. Nat. Mater..

[49] Guo N, Leu MC (2013). Additive manufacturing: technology, applications and research needs. Front. Mech. Eng..

[50] Abdulhameed O, Al-Ahmari A, Ameen W, Mian SH (2019). Additive manufacturing: challenges, trends, and applications. Adv. Mech. Eng..

[51] Halkjær S, Sigmund O, Jensen JS (2006). Maximizing band gaps in plate structures. Struct. Multidiscip. Optim..

[52] Elmadih W (2019). Three-dimensional resonating metamaterials for low-frequency vibration attenuation. Sci. Rep..

[53] Sheng-Bing C, Ji-Hong W, Gang W, Xiao-Yun H, Xi-Sen W (2011). Locally resonant gaps of phononic beams induced by periodic arrays of resonant shunts. Chin. Phys. Lett..

[54] Nobrega E, Gautier F, Pelat A, Dos Santos J (2016). Vibration band gaps for elastic metamaterial rods using wave finite element method. Mech. Syst. Signal Process..

[55] Tasch D, Mad A, Stadlbauer R, Schagerl M (2018). Thickness dependency of mechanical properties of laser-sintered polyamide lightweight structures. Addit. Manuf..

[56] ASTM, I. Standard test methods for flexural properties of unreinforced and reinforced plastics and electrical insulating materials. *ASTM D790-07* (2007).

